# Association between sleep duration and metabolic syndrome: a cross-sectional study

**DOI:** 10.1186/s12889-018-5557-8

**Published:** 2018-06-13

**Authors:** Claire E. Kim, Sangah Shin, Hwi-Won Lee, Jiyeon Lim, Jong-koo Lee, Aesun Shin, Daehee Kang

**Affiliations:** 1 Department of Preventive Medicine, Seoul National University College of Medicine, 102 Daehak-ro, Jongno-gu, Seoul, 03080 Korea; 2 Department of Biomedical Sciences, Seoul National University College of Medicine, Seoul, Korea; 3 Department of Food and Nutrition, Chung-Ang University, Seoul, Gyeonggi-do 17546 Korea; 4 JW Lee Center for Global Medicine, Seoul National University College of Medicine, Seoul, Korea; 5 Department of Family Medicine, Seoul National University Hospital, Seoul, Korea; 6 Institute of Environmental Medicine, Seoul National University Medical Research Center, Seoul, Korea

**Keywords:** Sleep duration, Diabetes, Metabolic syndrome, Metabolic disorder, Epidemiology

## Abstract

**Background:**

Both short and long sleep duration have been consistently studied as a risk factor for obesity, hyperglycemia and hypertension. In this cross-sectional study, we provide an updated analysis of the Health Examinees (HEXA) study on the association between sleep duration and metabolic syndrome (MetS) occurrence among Koreans age 40–69 year olds.

**Methods:**

A total of 133,608 subjects (44,930 men, 88,678 women) were enrolled in the HEXA study 2004–2013. Sleep duration was categorized into 4 sleep categories (< 6 h, 6 to < 8 h, 8 to < 10 h, ≥10 h). MetS criterion was based on the National Cholesterol Education Program, Adult Treatment Panel III. Logistic regression was used to calculate adjusted odds ratios (ORs) and 95% confidence intervals (CIs).

**Results:**

Compared with individuals sleeping 6 to < 8 h per day, less than 6 h of sleep was associated with MetS (multivariable adjusted OR: 1.12, 95% CI: 1.05–1.19) and elevated waist circumference (1.15, 1.08–1.23) among men; with elevated waist circumference (1.09, 1.04–1.14) among women. Greater than 10 h of sleep was associated with MetS (1.28, 1.08–1.50) and elevated triglycerides (1.33, 1.14–1.56) among men; with MetS (1.40, 1.24–1.58), elevated waist circumference (1.14, 1.02–1.27), elevated triglycerides (1.41, 1.25–1.58), reduced high-density lipoprotein cholesterol (HDL-C) (1.24, 1.12–1.38), and elevated fasting glucose (1.39, 1.23–1.57) among women.

**Conclusions:**

Less than 6 h of sleep is associated with elevated waist circumference among both men and women and with MetS among men only. Greater than 10 h of sleep is associated with MetS and elevated triglycerides among both men and women and with elevated waist circumference, reduced HDL-C, and elevated fasting glucose among women only.

**Electronic supplementary material:**

The online version of this article (10.1186/s12889-018-5557-8) contains supplementary material, which is available to authorized users.

## Background

Sleep is a lifestyle component that is often overlooked but can be viewed as an important preventive measure, an indicator to assess one’s current health status, or a health outcome which may lead to other health complications [1]. Accordingly, to promote good sleep quality, full daytime alertness and overall well-being, sleep guidelines have recommended adults to sleep 7–8 h per night and to practice healthy sleep hygiene such as limiting daytime naptimes to 30 min, avoiding stimulants such as caffeine before bedtime, avoiding heavy or rich foods (i.e. fatty or fried, spicy foods, citrus fruits) and ensuring adequate exposure to natural light [2, 3]. However, both insufficient and excessive sleep have been consistently reported to be associated with various health-related conditions such as hypertension, obesity [4], diabetes [5, 6], cardiovascular events or mortality [5–9], and stroke [5]. While many of these epidemiological studies can be summarized to show a U- shaped association between sleep duration and poor health outcomes, specifically how many hours are deemed deleterious and potential gender differences in the association remain equivocal [10, 11]. Additionally, many studies have been broadly defined by ‘short’ and ‘long’ sleep with varying sleep hour categories, which may cloud the dose-response relationship between the specific hours of sleep and health outcomes.

Metabolic syndrome (MetS) is defined as a metabolic disorder consisting of at least three of the following: elevated waist circumference, high triglyceride levels, low high-density cholesterol levels, hypertension and high fasting glucose. Given its high prevalence rates in Korea, approximately 25–30% among adults throughout the last decade, it is critical to identify the modifiable risk factors associated with metabolic syndrome and its components [12]. In Korea, the Health Examinees (HEXA) study provides information on sleep duration, a lifestyle factor that has been studied to be associated with metabolic syndrome. Specifically, a preliminary HEXA study on sleep duration and metabolic syndrome has been published to demonstrate an association between long sleep and metabolic syndrome among middle-aged and elderly Korean women [13]. However, this preliminary study was limited to HEXA survey years 2004–2008 which had categorized sleep duration into 2- h intervals, limiting specific sleep hour effects. Therefore, the current study, with an updated analysis of the previous HEXA study, present findings not shown in prior studies. This current study is the largest study examining a dose-response association between sleep duration (with supplemental analysis using 1-h interval sleep duration) and MetS and its components among both men and women.

## Methods

### Study population

The HEXA study (*n* = 169,722) is a large-scale genomic community-based study comprised of Koreans in age range 40–69 during the years 2004–2013. Details of the HEXA study rationale, design, and baseline characteristics are described in previous papers [14, 15]. This study uses HEXA-Gem (HEXA-G) participant sample that have been further restricted with participating site eligibility criteria [16].

**Fig. 1:**
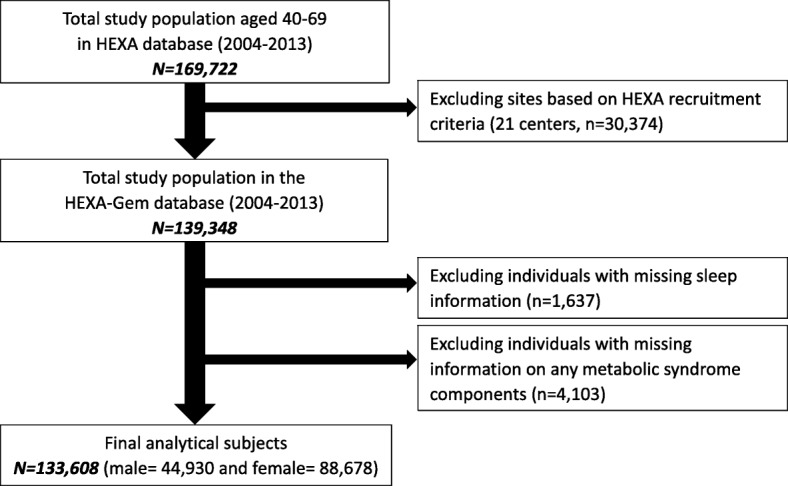
Flow diagram of analytical sample: the HEXA study (2004-2013)

Among the HEXA-G (*n* = 139,348) study subjects, participants with missing sleep information (*n* = 1637), or on any metabolic syndrome component (*n* = 4103) were excluded. A total of 133,608 subjects with 44,930 men and 88,678 women (Fig. 1) were included as the final analytic sample. The HEXA study protocol was approved by the Institutional Review Board (IRB) of the Seoul National University Hospital, Seoul, Korea (IRB number 0608–018- 179) and the Korea National Institute of Health (IRB number 2014–08-02-3C-A). All study participants gave written informed consent prior to entering the study.

### Assessment of sleep duration

For HEXA study years 2004–2008, sleep duration was assessed by the following question: “In the past year, on average, how many hours of sleep (including daytime naps) did you take per day?” with the following 4 category responses: < 6 h, 6 to < 8 h, 8 to < 10 h, ≥10 h. For HEXA study years 2009–2013, sleep duration question was modified to be open-ended with a slight modification in the question: “In the past year, on average, how many hours/minutes of sleep (including daytime naps) did you take per day?” The responses were then converted to the same 4 sleep category responses. In the multivariable analysis, 6 to < 8 h category was selected as reference since 6 to < 8 h is the median sleep category for the sample and to ensure comparability with the previous HEXA study [13].

### Definition of metabolic syndrome

The National Cholesterol Education Program Adult Treatment Panel III (NCEP ATP III) is a commonly used metabolic syndrome criteria oriented towards assessing cardiovascular diseases [17]. Our study defined metabolic syndrome using NCEP ATP III to ensure consistency and comparability with our previously published HEXA studies [18]. Participants who met three or more of the following criteria were classified as having metabolic syndrome: 1) waist circumference (WC) ≥90 and ≥ 80 cm for men and women, respectively; 2) triglycerides (TG) ≥150 mg/dL or drug treatment for elevated triglycerides; 3) high-density lipoprotein cholesterol (HDL-C) ≤40 and ≤ 50 mg/dL in men and women, respectively; 4) systolic blood pressure (BP) ≥130, diastolic BP ≥85 mmHg or drug treatment for elevated BP; and 5) fasting glucose ≥100 mg/dL or drug treatment for elevated fasting glucose.

### Covariates

The final model used in our study adjusted for the following covariates: age, education, occupation, marital status, and menopausal status (women only). Each of the covariates was categorized in the same way as the previously HEXA study on snoring and metabolic syndrome [18]. Education had three categories: middle school or below, high school graduate, and college or above. Occupation had three categories: non-manual, manual, and unemployed. Marital status had two categories: married or single. Menopausal status had two categories: pre- or post-menopausal.

Additional lifestyle covariates were considered. Current smokers were defined as those who smoked a minimum of 100 cigarettes during their lifetime and continued to smoke; non-smokers as those who have never smoked in their lifetime or have quit. Current drinkers were defined as those who drink alcohol at the time of survey and non-drinkers as those who have never drank alcohol or have abstained from alcohol drinking. Regular exercisers were defined as those engaging in routine physical activity. A food composition table proposed by the Korean Health and Industry of Development Institute was used to calculate the dietary intake measured by total caloric (k/cal) intake [19]. For all categorical covariates, missing data was assigned as “unknown”. Missing data was assigned as “unknown” for categorical variables.

### Statistical analysis

We’ve assess the association between sleep duration and MetS separately by gender. Likelihood ratio tests with the use of a cross-product term to calculate gender interaction *p*-values. To assess the basic characteristics of our sample in regards to sleep duration categories, a chi-square test (for categorical variables) and analysis of variance (ANOVA) (for continuous variables) were performed. A multivariable stepwise analysis was used to determine a parsimonious model for the final logistic regression models. To calculate prevalence odds ratios (ORs) and 95% confidence intervals (95% CIs), logistic regression analyses were performed. All *p*-values were two-sided, and statistical significance was set at below 0.05. A parsimonious model of regression was determined via multivariable stepwise analysis. The final model was adjusted for age (continuous), education, occupation, menopausal status (only women), smoking (only men) and drinking status, routine exercise and dietary intake (continuous) were adjusted. Marital status was not included in the models as it did not pose a significant effect on the relationship between sleep duration and MetS. While smoking is an important covariate for both sleep duration and MetS occurrence, the percent of current smokers among women was an average of 2.1% and therefore, was deleted from the model in women. Energy intake variable accounted for the individual dietary factors (i.e., fruit, vegetables, and meat) as they were highly correlated. A separate analysis with individual dietary factors adjusted did not affect the association between sleep duration and MetS. Moreover, we excluded subjects with a previous diagnosis of type 2 diabetes, hypertension, and dyslipidemia to account for comorbidities via sensitivity analysis. We also examined 1-h interval sleep duration and MetS and its components as a supplemental analysis. SAS software version 9.4 (SAS Institute, Cary, NC, USA) was used to perform all statistical analyses.

## Results

A summary of the sample baseline characteristics categorized by sleep duration is available in Table 1. About 10.9% of men and 12.7% of women slept less than 6 h, 1.5% of men and 1.7% of women slept greater than 10 h. The overall prevalence of MetS was 29.1% men and 24.5% women (Table 2). All selected covariates differed at statistical significance among the sleep duration categories.

**Table 1:** Baseline characteristics^a^ by sleep duration, the Health Examinees-Gem (HEXA-G) study 2004–2013

	Sleep Duration					
	Total	< 6 h	6 to < 8 h	8 to < 10 h	≥10 h	*p*-value^b^
Men, N	44,930	4906 (10.9)	29,141 (64.9)	10,213 (22.7)	670 (1.5)	
Age, years	53.6 ± 8.4	54.1 ± 8.5	53.0 ± 8.3	54.7 ± 8.3	56.6 ± 7.8	< 0.001
Education						< 0.001
Middle school or below	9672 (21.5)	1149 (23.4)	5461 (18.7)	2794 (27.4)	268 (40.0)	
High school graduate	18,390 (40.9)	1967 (40.1)	11,843 (40.6)	4301 (42.1)	279 (41.6)	
College or above	16,368 (36.4)	1726 (35.2)	11,511 (39.5)	3015 (29.5)	116 (17.3)	
Occupation						< 0.001
Non-manual	14,569 (32.4)	1573 (32.1)	10,311 (35.4)	2587 (25.3)	98 (14.6)	
Manual	20,960 (46.7)	2341 (47.7)	13,348 (45.8)	4933 (48.3)	338 (50.5)	
Unemployed	8440 (18.8)	897 (18.3)	4918 (16.9)	2411 (23.6)	214 (31.9)	
Married	42,187 (93.9)	4505 (91.8)	27,417 (94.1)	9643 (94.4)	622 (92.8)	< 0.001
Current smokers	18,732 (41.7)	2132 (43.5)	11,856 (40.7)	4486 (43.9)	258 (38.5)	0.009
Current drinkers	32,642 (72.7)	3434 (70.0)	21,539 (73.9)	7209 (70.6)	460 (68.7)	< 0.001
Regular exercisers	25,671 (57.1)	2642 (53.9)	17,073 (58.6)	5634 (55.2)	322 (48.1)	< 0.001
Dietary intake (k/cal)	1861.4 ± 562.6	1864.1 ± 591.3	1865.7 ± 552.2	1847.6 ± 571.4	1861.1 ± 651.2	0.048
Women, N	88,678	11,277 (12.7)	53,764 (60.6)	22,124 (24.9)	1513 (1.7)	
Age, years	52.3 ± 7.8	53.8 ± 7.7	52.0 ± 7.7	52.2 ± 7.9	52.2 ± 8.0	< 0.001
Education						< 0.001
Middle school or below	32,325 (36.5)	4842 (42.9)	18,414 (34.3)	8406 (38.0)	663 (43.8)	
High school graduate	38,159 (43.0)	4373 (38.8)	23,673 (44.0)	9488 (42.9)	625 (41.3)	
College or above	17,209 (19.4)	1905 (16.9)	11,128 (20.7)	3964 (17.9)	212 (14.0)	
Occupation						< 0.001
Non-manual	11,558 (13.0)	1356 (12.0)	7873 (14.6)	2232 (10.1)	97 (6.4)	
Manual	22,701 (25.6)	3296 (29.2)	14,124 (26.3)	4991 (22.6)	290 (19.2)	
Unemployed	52,185 (58.9)	6400 (56.8)	30,475 (56.7)	14,240 (64.4)	1070 (70.7)	
Married	76,725 (86.5)	9301 (82.5)	46,923 (87.3)	19,237 (87.0)	1264 (83.5)	< 0.001
Post-menopausal	50,306 (56.7)	7125 (63.2)	30,034 (55.9)	12,322 (55.7)	825 (54.5)	< 0.001
Current smokers	1896 (2.1)	311 (2.8)	970 (1.8)	555 (2.5)	60 (4.0)	< 0.001
Current drinkers	27,104 (30.6)	3358 (29.8)	16,642 (31.0)	6650 (30.1)	454 (30.0)	0.018
Regular exercisers	45,371 (51.2)	5384 (47.7)	27,961 (52.0)	11,312 (51.1)	714 (47.2)	< 0.001
Dietary intake (k/cal)	1711.6 ± 583.0	1674.0 ± 590.1	1714.1 ± 566.9	1721.7 ± 606.6	1753.6 ± 715.6	< 0.001

^a^Values are means ±SD or n (%)

^b^*p*-values for differences among sleep duration categories were calculated by chi-square tests for categorical variables and ANOVA for continuous variables

**Table 2:** Metabolic syndrome (MetS) prevalent cases^a^ by sleep duration, the Health Examinees-Gem (HEXA-G) study 2004–2013

	Sleep Duration					
	Total	< 6 h	6 to < 8 h	8 to < 10 h	≥10 h	*p*-value^c^
Men	44,930	4906	29,141	10,213	670	
MetS^b^	13,072 (29.1)	1536 (31.3)	8288 (28.4)	3010 (29.5)	238 (35.5)	< 0.001
WC ≥90 cm	13,190 (29.4)	1573 (32.1)	8438 (29.0)	2957 (29.0)	222 (33.1)	< 0.001
Serum TG ≥150 mg/dL	17,651 (39.3)	1926 (39.3)	11,293 (38.8)	4135 (10.5)	297 (44.3)	0.008
Serum HDL-C ≤ 40 mg/dL	10,346 (23.0)	1123 (22.9)	6606 (22.7)	2446 (24.0)	171 (25.5)	0.024
BP ≥130/85 mmHg	23,639 (52.6)	2656 (54.1)	15,227 (52.3)	5387 (52.8)	369 (55.1)	0.051
Fasting glucose ≥100 mg/dL	15,566 (34.7)	1731 (35.3)	9953 (34.2)	3624 (35.5)	258 (38.5)	0.009
Women	88,678	11,277	53,764	22,124	1513	
MetS^b^	21,754 (24.5)	3092 (27.4)	12,573 (23.4)	5622 (25.4)	467 (30.9)	< 0.001
WC ≥80 cm	36,877 (41.6)	5175 (45.9)	21,836 (40.6)	9168 (41.4)	698 (46.1)	< 0.001
Serum TG ≥150 mg/dL	20,296 (22.9)	2737 (24.3)	11,817 (22.0)	5303 (24.0)	439 (29.0)	< 0.001
Serum HDL-C ≤ 50 mg/dL	31,648 (35.7)	4056 (36.0)	18,765 (34.9)	8208 (37.1)	619 (40.9)	< 0.001
BP ≥130/85 mmHg	32,904 (27.1)	4593 (40.7)	19,479 (36.2)	8233 (37.2)	599 (39.6)	< 0.001
Fasting glucose ≥100 mg/dL	17,277 (19.5)	2422 (21.5)	10,044 (18.7)	4439 (20.1)	372 (25.0)	< 0.001

^a^Values are n (%)

^b^MetS: the presence of 3 or more of the following components: (1) elevated waist circumference (WC); (2) high triglyceride (TG) levels; (3) low high density lipoprotein–cholesterol (HDL-C) or taking anticholesterol medication; (4) high blood pressure (BP) or taking antihypertensive medicine; (5) high fasting glucose levels or taking medication to treat diabetes mellitus

^c^*p*-values for differences among sleep duration categories were calculated by chi-square tests

The odds ratios for MetS and its components by sleep duration are in Table 3. In both men and women, ORs displayed a J-shaped association between sleep duration and MetS (< 6 h OR: 1.12, 95% CI: 1.05–1.19 and ≥ 10 h OR: 1.28, 95% CI: 1.08–1.50 in men; < 6 h OR: 1.05, 95% CI: 1.00–1.10 and ≥ 10 h OR: 1.40, 95% CI: 1.24–1.58 in women). Less than 6 h sleep was also associated with elevated waist circumference (OR: 1.15, 95% CI: 1.08–1.23 in men; OR: 1.09, 95% CI: 1.04–1.14 in women). Among women, ≥10 h sleep was associated with all components of MetS, with the exception of elevated blood pressure. On the other hand, among the components, only elevated triglyceride levels were associated with ≥10 h sleep among men. Gender interaction was significant for MetS and all its components with p-interaction values < 0.001. Moreover, a sensitivity analysis accounting for did not alter the relationship of sleep duration with the odds for MetS (< 6 h OR: 1.10, 95% CI: 1.01–1.19, ≥10 h OR: 1.21, 95% CI: 0.97–1.51 in men and < 6 h OR: 1.00 95% CI: 0.94–1.07; ≥10 h OR: 1.42, 95% CI: 1.3–1.65 in women).

**Table 3:** Odds ratios (ORs)^a^ of metabolic syndrome (MetS) by sleep duration, the Health Examinees-Gem (HEXA-G) study 2004–2013

	Sleep Duration			
	< 6 h	6 to < 8 h	8 to < 10 h	≥10 h
Men^c^	4906	29,141	10,213	670
MetS^b^	1.12 (1.05–1.19)	Ref	1.01 (0.96–1.06)	1.28 (1.08–1.50)
WC ≥90 cm	1.15 (1.08–1.23)	Ref	0.98 (0.93–1.03)	1.15 (0.97–1.35)
Serum TG ≥150 mg/dL	1.03 (0.97–1.10)	Ref	1.09 (1.04–1.15)	1.33 (1.14–1.56)
Serum HDL-C ≤ 40 mg/dL	0.98 (0.91–1.05)	Ref	1.04 (0.98–1.10)	1.12 (0.94–1.34)
BP ≥130/85 mmHg	1.05 (0.98–1.11)	Ref	0.95 (0.91–1.00)	0.96 (0.82–1.12)
Fasting glucose ≥100 mg/dL	1.02 (0.96–1.09)	Ref	1.00 (0.95–1.05)	1.07 (0.91–1.26)
Women^c^	11,277	53,764	22,124	1513
MetS^b^	1.05 (1.00–1.10)	Ref	1.08 (1.04–1.12)	1.40 (1.24–1.58)
WC ≥80 cm	1.09 (1.04–1.14)	Ref	0.99 (0.96–1.02)	1.14 (1.02–1.27)
Serum TG ≥150 mg/dL	1.01 (0.97–1.07)	Ref	1.10 (1.05–1.14)	1.41 (1.25–1.58)
Serum HDL-C ≤ 50 mg/dL	0.96 (0.92–1.00)	Ref	1.08 (1.04–1.11)	1.24 (1.12–1.38)
BP ≥130/85 mmHg	1.03 (0.99–1.08)	Ref	1.01 (0.98–1.05)	1.11 (0.99–1.24)
Fasting glucose ≥100 mg/dL	1.07 (1.02–1.13)	Ref	1.07 (1.02–1.11)	1.39 (1.23–1.57)

^a^ORs adjusted for: age (continuous), education (middle school or below, high school graduate, college or above, unknown), occupation (non-manual, manual, unemployed, unknown), smoking (current, non, unknown; men only), menopausal status (pre, post, unknown; women only), alcohol drinking (current, non, unknown), regular exercise (yes, no, unknown), dietary intake (continuous)

^b^MetS: the presence of 3 or more of the following components: (1) elevated waist circumference (WC); (2) high triglyceride (TG) levels; (3) low high density lipoprotein–cholesterol (HDL-C) or taking anticholesterol medication; (4) high blood pressure (BP) or taking antihypertensive medicine; (5) high fasting glucose levels or taking medication to treat diabetes mellitus

^c^Gender p-interaction value < 0.001; interaction term was assessed by likelihood ratio tests with the use of a cross-product term

To assess the dose-response relationship of specific sleep duration hours and MetS, a supplemental analysis from HEXA study years 2009–2013 (73,530 subjects of which 24,979 men and 48,551 women) was performed (Additional file 1: Table S1). Sleep duration was categorized into 1-h intervals, from < 5 h to ≥10 h. Among men, only 5 h sleep was associated with metabolic syndrome (OR: 1.13, 95% CI: 1.02–1.25). In contrast, among women, 9 and ≥ 10 h sleep were significantly associated with MetS (OR: 1.15, 95% CI: 1.04–1.27 and OR: 1.37, 95% CI: 1.16–1.63 respectively). In the supplemental analysis, gender p-interaction value was significant for MetS and its components (all p-interaction < 0.001) but not for low HDL-C and high fasting glucose (p-interaction 0.303 and 0.323, respectively).

## Discussion

The results of the updated HEXA-G (2004–2013) analysis on sleep duration and metabolic syndrome and its components confirm and further expand on the previously published HEXA study (2004–2008) [13], displaying findings not shown in prior studies. In the previous HEXA study [13], after adjusting for covariates, 10 h sleep or greater was associated with MetS in women only (OR: 1.53, 95% CI: 1.32–1.78 for women; OR: 1.19, 95% CI: 0.98–1.46 for men); while, less than 6 h sleep was not associated with MetS in both men and women (OR: 1.09, 95% CI: 0.99–1.19; OR: 1.04, 95% CI: 0.97–1.11 respectively). However, in the current study, with expanded sample size and power, a positive association was observed between 10 h sleep or greater and MetS in both men and women (OR: 1.28, 95% CI: 1.08–1.50; OR: 1.40, 95% CI: 1.24–1.58 respectively) as well as in less than 6 h sleep among men (OR: 1.12, 95% CI: 1.05–1.19). In the supplemental analysis, a similar J-shape trend existed but with a significant positive association between 10 h sleep or greater and MetS only in women; between 5 h sleep and MetS only in men. Gender interaction in the association between sleep duration and metabolic syndrome was statistically significant in our study which complements the gender difference reported in a study looking at the association between sleep duration and mortality [20]. While the exact mechanisms are unclear, one explanation may be that women experiencing menopausal transition face erratic fluctuations and eventual decline in estrogens as well as ovarian oestradiol which may lead to frequent sleep disruptions [21, 22], a common characteristic of long sleep duration [23]. Another study posits that women may have shorter circadian period contributing to higher prevalence of insomnia and/or perception of less restorative sleep [24]. Additionally, a study examining the association between inflammatory markers and sleep duration observed higher levels of interleukin-6 (IL-6) and C-reactive protein (CRP) in women who slept less than 5 h or more than 9 h, while no significant marker variation was observed in men [25]. Notably, a recent meta-analysis stated that women may be more vulnerable to the effects of sleep disturbance and displayed greater increases of CRP and IL-6 compared with men. The review also reported that long sleep duration, but not short duration was associated with increases in CRP and IL-6 [26].

Few studies have reported gender-stratified sleep association with MetS. A meta-analysis of 12 cross-sectional and 3 cohort studies from North America, Europe, and Asia, has found that both less than 5 h and greater than 8 h sleep duration were associated with MetS but reported no gender differences between the association [27]. Additionally, a study in Korea reported that both short (less than or equal to 5 h) and long (greater than or equal to 9 h) sleep are related to increased risk of MetS, however, with gender adjusted [28]. Other studies broadly categorized hours of sleep into “short” and “long” and did not report the association between hour-specific sleep duration and MetS. For example, one cross-sectional study conducted in China categorized sleep duration into 2- h intervals and found that both short (less than 6 h) and long (greater than 9 h) sleep was associated with MetS in males only [29]. Similarly, a prospective study conducted in Korea has also used 2-h sleep intervals and reported that only short (less than 6 h) sleep was associated with MetS in a mixed gender population [30]. Furthermore, while a recent meta-analysis reported that a dose-response relationship exists between short sleep and MetS, it did not support the notion that long sleep is associated with MetS [31]. The opposite was observed in a study conducted in Korea in which greater than or equal to 9 h was associated with MetS but not with sleep less than or equal to 5 h [32].

Although the biological mechanism of sleep duration and MetS remains unclear, several potential endocrinologic, immunologic, and metabolic processes have been reported. Sleeping less than 7 h may cause reciprocal changes in circulating levels of leptin and ghrelin [33] which would increase appetite, caloric intake, reduce energy expenditure [34] facilitating an increase in waist circumference as well as overall obesity development. It may also cause impaired glycemic control (lowering glucose tolerance and thyrotropin concentration levels) increasing risk for hypertension and diabetes [35]. Other endocrinologic effects include increased cortisol levels which may elevate fasting glucose levels [36]. Additionally, clinical studies have shown that sleep deprivation results in increased levels of high-sensitivity CRP and IL-6 during, markers that have also been associated with constituents of MetS [37].

Likewise, number of studies report detrimental health effects of long sleep [27, 38] and suggest sleeping in moderation (approximately 7 h) rather than in abundance for optimum health [39]. Potential effects of long sleep include: increased sleep fragmentation with lower sleep quality [23], greater fatigue [40], limited photoperiod and greater physiological deprivation (i.e. exercise) [23]. All of these conditions are studied to be associated with insulin resistance, dyslipidemia and hormonal imbalance [41] which may lead to premature death [23, 39].

While the current study displays a correlation between sleep duration and MetS, there are a couple factors to consider. First, the current study is cross-sectional and therefore, causality between sleep duration and MetS cannot be construed. However, we’ve examined the association of baseline sleep duration with MetS incidence through an incidence analysis among the HEXA-G subjects who have completed the follow-up survey from 2012 to 2015 (54,504 subjects of which 18,522 men and 35,982 women). We found in both men and women who sleep more than 10 h, there was a marginal increased risk of MetS compared to those sleeping 6 to < 8 h (Hazard Ratio (HR): 1.18, 95% CI: 0.88–1.59 in men; HR: 1.19, 95% CI: 0.97–1.46 in women). Although not statistically significant, a prospective cohort study design with the total HEXA-G sample’s sleep duration and risk of MetS are warranted to support these exploratory findings. Second, sleep duration was assessed through self-report questionnaire instead of objective measures via the use of an actigraph or polysomnography. Therefore, it is important to note that ‘sleep duration’ may be reflective of ‘time in bed’, actual time spent asleep, or even how much sleep one believes was attained [42]. Nevertheless, studies have reported that self-report sleep has a moderate correlation (Pearson’s *p* = 0.31–0.47) to objectively assessed time spent asleep [43, 44] and hence, remains as a useful tool in large epidemiological studies. Third, total sleep time measured may include both nighttime sleep as well as naptime. Daytime napping behavior has been associated with lower sleep efficiency, shorter sleep duration, and consequently cardiovascular risk factors [45]. Hence, it would be informative to make the distinction between naptime and nighttime to separately assess their impact on health. Fourth, no comprehensive data on sleep quality/disturbances was available for analysis. Studies have reported associations between sleep disturbances and cardiovascular and metabolic disorders [38], which point to the importance of including sleep quality/disturbance to assess the effect of sleep on overall health. Fifth, the covariates such as smoking, alcohol drinking, and physical activity were included in the final model as categorical variables. Given that smoking, alcohol drinking, and physical activity are studied to be dose-dependent to health outcomes, there may be residual confounding effect that is not accounted for. Additionally, our study included menopausal status as a binary variable and does not include information on women experiencing menopausal transition, which has been studied to be a contributing factor to sleep patterns in women [22].

Despite these limitations, the current study is the largest study providing dose-response association between sleep duration and metabolic syndrome and its components. Using the HEXA-G database allowing for greater internal validity as well as additional robust subgroup analyses: the sample became more homogenous and the number of women and men have almost doubled from the previous study which gave more power to detect the associations between sleep and MetS that were unnoticed before. Furthermore, with the addition of extended HEXA study years from 2009 to 2013, hour-specific dose-response association was analyzed which highlighted the gender differences in association between sleep and MetS.

## Conclusions

In conclusion, after adjusting for covariates such as sociodemographic and lifestyle factors, sleep duration displayed an association with MetS and its components among both men and women. Gender differences were observed in regards to the effect of short and long sleep and their association with MetS-men were affected more by short sleep and women with long sleep. Further prospective studies using multiple measurements of sleep duration (i.e. sleep diaries and actigraphs) are warranted to assess the casual relationship between sleep duration and MetS and its components.

## Additional file

Additional file 1: **Table S1.** Odds ratios (ORs)^a^ of metabolic syndrome by sleep duration, the Health Examinees-Gem (HEXA-G) study 2009-2013^b^. (DOCX 32 kb)

## Abbreviations

95% CIs: 95% confidence intervals
ANOVA: Analysis of variance
BP: Blood pressure
CRP: C-reactive protein
HDL-C: High-density lipoprotein cholesterol
HEXA: Health examinees
HEXA-G: HEXA-Gem
IL-6: Interleukin-6
MetS: Metabolic syndrome
NCEP ATP III: National Cholesterol Education Program Adult Treatment Panel III
ORs: Odds ratios
TG: Triglycerides
WC: Waist circumference
