# Supplementary material for: Association between sleep duration and metabolic syndrome: a cross-sectional study
Source: BMC Public Health. 2018 Jun 13;18:720. doi: 10.1186/s12889-018-5557-8 (PMC5998453; doi:10.1186/s12889-018-5557-8)
Supplement: Supplementary file 1 — Table S1. Odds ratios (ORs)a of metabolic syndrome by sleep duration, the Health Examinees-Gem (HEXA-G) study 2009-2013b. (DOCX 32 kb) [file 12889_2018_5557_MOESM1_ESM.docx]

**Table S1.** Odds ratios (ORs)^a^ of metabolic syndrome by sleep duration, the Health Examinees-Gem (HEXA-G) study 2009-2013^b^

|  | **Sleep Duration** | | | | | | |
| --- | --- | --- | --- | --- | --- | --- | --- |
|  | **<5 hrs** | **5 hrs** | **6 hrs** | **7 hrs** | **8 hrs** | **9 hrs** | **≥10 hrs** |
| *Men^d^, N= 24,979* | *606* | *2,242* | *6,967* | *8,968* | *4,775* | *1,078* | *343* |
| MetS^c^ | 1.09 (0.91-1.31) | 1.13 (1.02-1.25) | 1.00 (0.94-1.08) | Ref | 0.99 (0.92-1.07) | 1.04 (0.90-1.19) | 1.18 (0.94-1.49) |
| WC ≥90 cm | 1.11 (0.92-1.33) | 1.23 (1.11-1.36) | 1.09 (1.02-1.17) | Ref | 0.99 (0.91-1.07) | 0.96 (0.83-1.11) | 1.00 (0.79-1.28) |
| Serum TG ≥150 mg/dL | 1.04 (0.88-1.23) | 0.97 (0.88-1.07) | 0.96 (0.90-1.02) | Ref | 1.06 (0.98-1.14) | 1.08 (0.94-1.23) | 1.32 (1.06-1.65) |
| Serum HDL-C ≤40 mg/dL | 0.95 (0.78-1.17) | 0.97 (0.86-1.08) | 1.00 (0.93-1.08) | Ref | 1.05 (0.97-1.14) | 1.26 (1.09-1.46) | 1.05 (0.82-1.36) |
| BP ≥130/85 mmHg | 1.05 (0.88-1.24) | 1.05 (0.96-1.16) | 1.09 (1.02-1.16) | Ref | 0.96 (0.89-1.03) | 0.93 (0.81-1.06) | 1.08 (0.86-1.35) |
| Fasting glucose ≥100 mg/dL | 1.11 (0.93-1.32) | 1.03 (0.94-1.14) | 0.94 (0.88-1.01) | Ref | 0.99 (0.92-1.07) | 1.04 (0.91-1.19) | 1.03 (0.82-1.29) |
|  |  |  |  |  |  |  |  |
| *Women^d^, N= 48,551* | *1,496* | *4,767* | *12,990* | *16,336* | *9,977* | *2,271* | *714* |
| MetS^c^ | 1.11 (0.98-1.26) | 1.05 (0.97-1.13) | 1.02 (0.97-1.08) | Ref | 1.05 (0.99-1.12) | 1.15 (1.04-1.27) | 1.37 (1.16-1.63) |
| WC ≥80 cm | 1.11 (0.99-1.24) | 1.15 (1.07-1.23) | 1.07 (1.02-1.12) | Ref | 0.97 (0.92-1.03) | 1.09 (0.99-1.20) | 1.17 (1.00-1.37) |
| Serum TG ≥150 mg/dL | 1.11 (0.99-1.24) | 1.15 (1.07-1.23) | 1.07 (1.02-1.12) | Ref | 0.97 (0.92-1.03) | 1.09 (0.99-1.20) | 1.17 (1.00-1.37) |
| Serum HDL-C ≤50 mg/dL | 0.99 (0.88-1.11) | 0.93 (0.86-0.99) | 1.00 (0.95-1.05) | Ref | 1.05 (1.00-1.11) | 1.19 (1.09-1.31) | 1.27 (1.08-1.48) |
| BP ≥130/85 mmHg | 1.11 (0.99-1.25) | 1.05 (0.98-1.13) | 1.06 (1.00-1.11) | Ref | 1.03 (0.97-1.08) | 1.04 (0.94-1.14) | 1.15 (0.98-1.35) |
| Fasting glucose ≥100 mg/dL | 1.13 (0.99-1.29) | 1.09 (1.01-1.19) | 0.99 (0.94-1.06) | Ref | 1.04 (0.98-1.11) | 1.10 (0.98-1.22) | 1.22 (1.02-1.46) |

^a^ORs adjusted for: age (continuous), education (middle school or below, high school graduate, college or above, unknown), occupation (non-manual, manual, unemployed, unknown), smoking (current, non, unknown; men only), menopausal status (pre, post, unknown; women only), alcohol drinking (current, non, unknown), regular exercise (yes, no, unknown), dietary intake (continuous). ^b^ HEXA-G 2004-2008 participants were excluded as their sleep survey was limited to 4 sleep duration category responses.
^c^ MetS: the presence of 3 or more of the following components: (1) elevated waist circumference (WC); (2) high triglyceride (TG) levels; (3) low high density lipoprotein–cholesterol (HDL-C) or taking anticholesterol medication; (4) high blood pressure (BP) or taking antihypertensive medicine; (5) high fasting glucose levels or taking medication to treat diabetes mellitus.
^d^ Gender p-interaction value for MetS and its components <0.001, with the exception of low HDL-C and high fasting glucose (p-interaction 0.303 and 0.323, respectively); interaction term was assessed by likelihood ratio tests with the use of a cross-product term
